# Severity of Pain at Admission and Development of Symptoms of Anxiety and Depression: A Study of Burn Patients at a Tertiary Healthcare Facility in Ghana

**DOI:** 10.7759/cureus.82034

**Published:** 2025-04-10

**Authors:** Robert Djagbletey, George Aryee, Veronica M Aborbi, Raymond Essuman, Janet Pereko, Joycelyn K Vogelsang, Esther Brobbey, Ebenezer Owusu Darkwa

**Affiliations:** 1 Anesthesia, Intensive Care and Pain Management, University of Ghana Medical School, Accra, GHA; 2 Public Health, Korle-Bu Teaching Hospital, Accra, GHA; 3 Plastic and Reconstructive Surgery, Korle-Bu Teaching Hospital, Accra, GHA; 4 Psychiatry, Korle-Bu Teaching Hospital, Accra, GHA; 5 Respiratory Therapy, University of Ghana School of Biomedical and Allied Health Sciences, Accra, GHA

**Keywords:** anxiety, burn, depression, fire, injury, pain

## Abstract

Background

Burns remain a global public health concern and one of the major causes of painful injury, which impacts patients physically and psychologically. Pain causes more suffering in the acute stage and rehabilitation in burn patients, which is associated with anxiety, depression, and post-traumatic stress disorder that can lead to long-term consequences, which negatively affects the quality of life (QoL) of the patient.

Objective

This study aimed to determine the influence of the severity of pain at admission on the development of symptoms of anxiety and depression among burn patients within the first week of admission.

Methods

An analytical cross-sectional study was conducted among adult burn patients at the Burns Centre of the Korle-Bu Teaching Hospital. Patients’ demographic and clinical characteristics, such as age, cause of burns, degree of burns, and percentage of total body surface area (TBSA), were recorded. Hospital Anxiety and depression scale (HADS) was used to assess anxiety and depression symptoms, while the Visual Analog Scale (VAS) was used to evaluate the intensity of pain. Simple linear regression was used to determine the influence of the severity of pain at admission on symptoms of anxiety and depression.

Results

Sixty-five adult inpatients were enrolled, with a mean age of 35.9 years. About 92% presented with severe pain, and two-thirds developed severe anxiety (66.2%) or depressive (67.7%) symptoms. The mean (±SD) anxiety symptoms, depression symptoms, and pain scores were 11.4(±2.7), 11.8(±3.4), and 8.5(±1.6), respectively. Mean anxiety symptom scores were significantly different between the age groups, %TBSA, and severity of pain. Mean depression symptom scores were significantly different between the severity of pain and the cause of burn injury. There was a significant positive relationship between the severity of pain on admission and the level of depression and anxiety symptoms.

Conclusion

Patients with burn injury present with severe pain at admission, and the majority develop significant anxiety and depressive symptoms within the first week of admission. The intensity of pain at admission is significantly associated with the level of anxiety and depression.

## Introduction

Burns remain a global public health concern and one of the major causes of painful injury worldwide, affecting all age groups. The World Health Organization (WHO) estimates that the lifetime incidence of severe burns is 1% and is ranked fourth among all injuries [1,2].

Burns greatly affect not just the physical but also the psychological well-being of patients. Common psychological problems experienced by burn injury patients include anxiety, depression, post-traumatic stress disorder, concern about bodily disfigurement, social isolation, and financial burden due to the prolonged duration of hospitalization and treatment required [3].

Several factors, including pain intensity, the depth/degree of burns, and percentage total body surface area (%TBSA) burned, have been noted to influence the incidence and severity of post-burn psychological symptoms [4-6]. High pain scores are associated with the development of more severe anxiety and depression among patients with severe burn within two weeks of their burn trauma [7]. Patients who have had prolonged and painful treatment for burn injuries and those who experienced high anxiety levels as a result of pain have been reported to be at risk of developing post-traumatic stress disorder long after hospital discharge [8].

Depression is not uncommon following burn injury [9]. Studies indicate that as much as 18%-45% of burn survivors experience moderate to severe depression long after their injuries have healed [10-12]. Though most studies do not find any gender difference with respect to the development of depression in burn patients, a few studies have reported that females with disfiguring facial injuries were at more risk of developing depression [7].

Not equipping burn patients with effective and appropriate coping skills predisposes them to developing long-term psychological disorders. However, prompt identification of pain-related anxiety and depression is important for early intervention to reduce the risk of developing other psychological symptoms and improve overall quality of life. The aim of this study was to determine the influence of the severity of pain at admission on the development of anxiety and depressive symptoms among burns patients within the first week of admission at a tertiary healthcare facility in Ghana.

## Materials and methods

Study design and site

This was an analytical cross-sectional study of adult patients admitted to the Burns Centre of the Korle-Bu Teaching Hospital (KBTH) between September 2020 and April 2021. The KBTH is a tertiary referral center in Ghana and the third-largest hospital in West Africa. The Burns Centre of the hospital has a capacity of 68 beds and sees an average of 320 patients weekly on an outpatient department (OPD) basis, of which about 30 are burn patients.

Inclusion and exclusion criteria

Burn patients aged 18 years and above who were admitted to the Burns Centre of the KBTH and consented were recruited into the study. Patients with a previous history of psychological or psychiatric disorders were excluded from the study.

Sample size determination

About 70% of burn patients have been found to experience moderate-to-severe symptoms of depression [3]. Using the Cochrane sample size determination formula, a sample size of 65 was found to be adequate.

Data collection

Patients admitted to the Burns Centre of the KBTH during the study period who satisfied the inclusion criteria and consented to the study were consecutively enrolled. Patients’ demographic characteristics such as age, sex, marital status, and occupation were obtained. Percentage of TBSA (%TBSA) burnt, cause of injury (electrical, chemical, scalds, and fire), degree of burn (first, second, and third degree), and severity of pain at admission were recorded. Patients were assessed for symptoms of anxiety and depression within the first week of admission. The severity of pain was assessed by the Pain Specialist using the Visual Analog Scale (VAS), and symptoms of anxiety and depression were assessed using the Hospital Anxiety and Depression Scale (HADS). VAS pain scores were classified as no pain (0), moderate pain (1-5), and severe pain (6-10). HADS scores were categorized as normal (0-7), borderline (8-10), and abnormal (11-21).

Data analysis

Data was entered in Microsoft Excel 2016 (Microsoft, Redmond, Washington), cleaned, and exported to IBM SPSS® (version 25; IBM Corp., Armonk, NY) for analysis. Categorical variables of the demographic characteristics, such as marital status, sex, and occupation, were summarized as proportions. Continuous variables such as age, pain score, and HADS score were summarized as means (±Sd). Linear regression was used to determine the factors associated with depression and anxiety symptoms. A p-value less than 0.05 was considered statistically significant.

Ethical considerations

Ethical approval was obtained from the Institutional Review Board of the KBTH (Protocol ID: KBTH-IRB/00032/2020). The study was explained to the patients, and informed consent was obtained before being enrolled in the study. Participation in this study was voluntary, and patients’ refusal did not affect their subsequent management.

## Results

A total of 65 participants were enrolled, with the majority being male, 38 (58.5%), and their mean (±Sd) age was 35.9 (±14.6) years. The majority of the patients, 47 (72.3%), were between the ages of 18 and 39 years, with only six (9.2%) being 60 years and older (Table 1).

**Table 1:** Demographic and burn-related characteristics ^a^Mean(±standard deviation)

Variable	Descriptive
Sex	
Male	38(58.5)
Female	27(41.50
Age	35.9 (±14.6)^ a^
18-39	47(72.3)
40-59	12(18.5)
≥60	6(9.2)
%TBSA	
<10	10(15.4)
10-20	36(55.4)
21-30	15(23.1)
>30	4(6.1)
Degree of burn	
First degree	38(58.5)
Second degree	22(33.8)
Third degree	5(7.7)
Cause of burn	
Fire	24(36.9)
Electrical	6(9.2)
Scalds	25(38.5)
Chemicals	10(15.4)
Severity of pain	8.5(±1.6)^ a^
Moderate (1-5)	5(7.7)
Severe (6-10)	60(92.3)
Anxiety	11.4(±2.7)^a^
Normal (0-7)	7(10.8)
Borderline case (8-10)	15(23.1)
Abnormal case (11-21)	43(66.2)
Depression	11.8(±3.4)^ a^
Normal (0-7)	2(3.1)
Borderline case (8-10)	19(29.2)
Abnormal case (11-21)	44(67.7)

Three-quarters of the patients had thermal burns. Nearly two-thirds presented with first-degree burns, with only five (7.7%) presenting with third-degree burns. More than half, 36 (55.4%), had burns with a TBSA between 10% and 20%, while only four (6.2%) had more than 30% TBSA burns. A significant number of the patients, 60 (92.3%), experienced severe pain, whilst 7.7% had moderate pain. The mean (±SD) anxiety symptoms, depression symptoms, and pain scores were 11.4 (±2.7), 11.8 (±3.4), and 8.5 (±1.6), respectively. Furthermore, 44 (67.7%) and 43 (66.2%) experienced abnormal depression and anxiety symptoms, respectively (Table 1).

There was a significant positive relationship between age, TBSA, and severity of pain and anxiety, while depression had a significant positive relationship with severity of pain. Severity of pain on admission and symptoms of depression and anxiety within the first week of admission yielded a regression coefficient of 0.616 (95% CI = 0.12‒1.12) with R-squared (R^2^) of 29.5% and 0.506 (95% CI=0.10‒0.91) with R^2^ of 30.0%, respectively (Table 2).

**Table 2:** Burn-related characteristics associated with anxiety and depression *Statistically significant (p-value <0.05), **Dummy variable reference, β(regression coefficient)

Variable	Anxiety		Depression	
	β	P-value	β	P-value
Age	0.035	0.042*	0.101	0.098
%TBSA	0.126	0.003*	0.134	0.095
Severity of pain	0.506	0.015*	0.616	0.017*
Cause of burns				
Fire	-0.512	0.234	-0.423	0.573
electrical	0.314	0.320	-0.206	0.863
Chemicals	0.143	0.567	1.490	0.139
Scalds **				

## Discussion

Burn injury remains a significant public health problem, and it affects all age groups worldwide. Women and children have been found to be the most at risk of burns, with scalds being the most common cause of burns in this group. This has been attributed mostly to injuries sustained from accidents in the kitchen [13]. In this study, the majority of patients were young adult males. The mean age of patients was 35.9 years, and flame was the most common cause of burns. Findings were similar to those by Abarca et al. [14].

The nature and intensity of pain associated with burns are influenced by several factors, including the size, extent, and location of the burn [15]. In this study, the majority of the patients sustained burns > 10 TBSA (84.6%), which could explain why most patients (92.1%) reported severe pain at admission, a result comparable to that by Prasad et al. [16].

Pain has both sensory and affective dimensions [17]. Major central nervous system areas involved in pain processing and modulation are also associated with pain and are known to be associated with anxiety and depression. A neuroinflammatory response with elevated central nervous system and serum inflammatory cytokines has been found to accompany not just pain but also anxiety and depression [18]. These may explain the inter-relationship between pain, anxiety, and depression.

Anxiety and depression have been reported as the most common psychological problems experienced by burn patients [19]. The severity of anxiety and depression among burn patients is related to the severity of pain [7], TBSA [20], and the degree of burns [3].

Aside from anxiety and depression, burn patients face other psychological problems such as post-traumatic stress disorder, concern about bodily disfigurement, social isolation, and the financial burden due to the prolonged duration of hospitalization and treatment required [3].

In this study, the majority of patients had high levels of anxiety and depressive symptoms. These symptoms could lead to psychological distress with resultant immediate to lasting negative impact on the well-being, function, and quality of life of the patients [21].

In this study, age, TBSA, and severity of pain were independent predictors of anxiety, while depression had severity of pain as an independent predictor. Chronic pain, anxiety, and depression are known to co-occur [22]. The long-term interrelationship between post-burn pain, anxiety, and depression has also been reported by Farzan et al. [23]. Locar et al. [7] demonstrated anxiety and depressive symptomatology among burns patients two weeks into admission. Our study sought to investigate if a relationship existed between the severity of pain on admission, anxiety, and depressive symptoms within the first week of admission.

Consistent with reports from studies [3,7,24], done after two weeks of admission, we found severity of pain at admission to be significantly related to both anxiety and depression symptoms. Severity of pain at admission was a significant predictor of the level of anxiety and depression symptoms burn patients developed within the first week of admission. A unit increase in severity of pain was associated with a 51% increase in the level of anxiety and a 62% increase in the level of depression among the burn patients.

Anxiety may arise from fear of injury to or loss of body parts or function, fear of loss of approval, or fear of inability to work/loss of job. In burn patients, depression may result from pain, the fears associated with anxiety, loss of income, and social isolation due to hospitalization [7].

Increased sympathetic stimulation, catecholamine, and proinflammatory cytokines released due to the stress response from burn injury may increase pain [25]. Pain from burn injury and management modalities and worry arising from the uncertainty of treatment and outcomes cause anxiety. This leads to a vicious circle of anxiety-pain-anxiety, which may cause depression.

The interplay between burn pain, neuroinflammation, and the emergence of anxiety and depression symptoms can be attributed to shared neurobiological mechanisms involving nociceptive signaling and inflammatory processes. Following a burn injury, the nociceptive system becomes highly sensitized due to the release of inflammatory mediators such as prostaglandins, cytokines (e.g., IL-1β, IL-6, TNF-α), and neuropeptides including substance P and calcitonin gene-related peptide (CGRP). These substances not only amplify pain perception but also contribute to central sensitization by activating microglia and astrocytes in the spinal cord and brain [26]. Pain-induced stress may exacerbate HPA axis dysregulation, elevating glucocorticoid levels, which impairs neurogenesis and synaptic plasticity, processes vital for emotional regulation [27]. The persistent activation of nociceptive pathways and overlapping neurochemical changes between pain and mood disorders underscore the biopsychosocial complexity of burn recovery. Clinically, the co-occurrence of chronic burn pain with psychiatric symptoms often results in a feedback loop, where psychological distress intensifies pain perception, which in turn worsens emotional well-being [8]. These findings advocate for an integrated treatment approach that concurrently targets pain and neuropsychiatric symptoms to improve rehabilitation outcomes for burn survivors.

The management of burns, therefore, requires a multi-disciplinary team from the time of admission to rehabilitation. Early involvement of pain and mental health specialists should be considered to afford the effective management of burn pain and injury, pain associated with burn treatment, as well as prompt recognition and management of psychological problems to improve well-being, quality of life, and prevent long-term psycho-social sequelae.

Understanding the interrelation of burn pain with anxiety and depression necessitates a multidimensional framework, such as the biopsychosocial model, which posits that biological, psychological, and social factors interact to shape the individual pain experience. Burn injuries not only elicit nociceptive and inflammatory responses but also invoke profound psychological stress and social disruptions that can intensify perceived pain and emotional distress. Within this framework, the pain-anxiety-depression (PAD) triad offers a more specific lens, highlighting the bidirectional and reinforcing nature of these conditions [28]. The shared neural circuitry and neurotransmitter systems - including the anterior cingulate cortex, amygdala, and the serotonergic and noradrenergic systems - underpin the overlap between nociception and affective disorders [29]. Furthermore, maladaptive cognitive processes such as catastrophizing and hypervigilance to pain contribute to increased emotional reactivity and reduced pain thresholds [30]. Social determinants, such as isolation, stigma from burn-related disfigurement, and reduced support networks, further exacerbate psychological burden and may hinder recovery. Integrating these models underscores the importance of early, holistic interventions that address both physical and emotional components of burn recovery, including multimodal pain management, psychological counseling, and social reintegration strategies. By recognizing the dynamic interplay among pain, mood, and psychosocial factors, clinicians can tailor treatment plans that mitigate chronicity and improve long-term functional outcomes in burn survivors.

We did not exclude patients with preexisting chronic pain disorders who may have had varying levels of undiagnosed anxiety and depression, and this could have impacted our findings. The findings of this study only show a relationship between the severity of burn pain on admission and the level of anxiety and depression developed by patients. This finding should be interpreted with caution as the cross-sectional design of the study does not allow for the establishment of a cause-and-effect relationship.

## Conclusions

Young male patients are more likely to present with burn injuries than female patients. A significant proportion presents with first to second-degree burns, and flame was the commonest cause of injury. A significant proportion presents with %TBSA greater than 10. The majority of patients present with severe pain at admission, and the majority develop significant anxiety and depressive symptoms within the first week of admission. There was a significant positive relationship between age, TBSA, and severity of pain and anxiety, while depression had a significant positive relationship with severity of pain. The severity of pain at admission was significantly associated with the level of anxiety and depression symptoms burn patients developed within the first week of admission.

## References

[1] Li H, Yao Z, Tan J, Zhou J, Li Y, Wu J, Luo G (2017). Epidemiology and outcome analysis of 6325 burn patients: a five-year retrospective study in a major burn center in Southwest China. Sci Rep.

[2] Wardhana A, Basuki A, H ADHP, Rizkita DN, Andarie AA, Canintika AF (2017). The epidemiology of burns in Indonesia ’ s national referral burn center from 2013 to 2015. Burn Open.

[3] Jain M, Khadilkar N, De Sousa A (2017). Burn-related factors affecting anxiety, depression and self-esteem in burn patients: an exploratory study. Ann Burns Fire Disasters.

[4] Thombs BD, Haines JM, Bresnick MG, Magyar-Russell G, Fauerbach JA, Spence RJ (2007). Depression in burn reconstruction patients: symptom prevalence and association with body image dissatisfaction and physical function. Gen Hosp Psychiatry.

[5] Pérez Boluda MT, Morales Asencio JM, Carrera Vela A (2016). The dynamic experience of pain in burn patients: a phenomenological study. Burns.

[6] Xie B, Xiao SC, Zhu SH, Xia ZF (2012). Evaluation of long term health-related quality of life in extensive burns: a 12-year experience in a burn center. Burns.

[7] Loncar Z, Bras M, Mickovic V (2006). The relationships between burn pain, anxiety and depression. Coll Anthropol.

[8] Van Loey NE, Van Son MJ (2003). Psychopathology and psychological problems in patients with burn scars: epidemiology and management. Am J Clin Dermatol.

[9] Fauerbach JA, McKibben J, Bienvenu OJ (2007). Psychological distress after major burn injury. Psychosom Med.

[10] Thombs BD, Bresnick MG, Magyar-Russell G, Lawrence JW, McCann UD, Fauerbach JA (2007). Symptoms of depression predict change in physical health after burn injury. Burns.

[11] Pall N, Künsebeck HW, Noah EM (2003). Psychosocial adjustments 5 years after burn injury. Burn.

[12] Wiechman SA, Ptacek JT, Patterson DR, Gibran NS, Engrav LE, Heimbach DM (2001). Rates, trends, and severity of depression after burn injuries. J Burn Care Rehabil.

[13] Peck MD (2011). Epidemiology of burns throughout the world. Part I: distribution and risk factors. Burns.

[14] Abarca L, Guilabert P, Martin N, Usúa G, Barret JP, Colomina MJ (2023). Epidemiology and mortality in patients hospitalized for burns in Catalonia, Spain. Sci Rep.

[15] Hanafiah Z, Potparic O, Fernandez T (2008). Addressing pain in burn injury. Curr Anaesth Crit Care.

[16] Prasad A, Thode HC Jr, Sandoval S, Singer AJ (2019). The association of patient and burn characteristics with itching and pain severity. Burns.

[17] Talbot K, Madden VJ, Jones SL, Moseley GL (2019). The sensory and affective components of pain: are they differentially modifiable dimensions or inseparable aspects of a unitary experience? A systematic review. Br J Anaesth.

[18] De La Rosa JS, Brady BR, Ibrahim MM, Herder KE, Wallace JS, Padilla AR, Vanderah TW (2024). Co-occurrence of chronic pain and anxiety/depression symptoms in U.S. adults: prevalence, functional impacts, and opportunities. Pain.

[19] Lawrence JW, Mason ST, Schomer K, Klein MB (2012). Epidemiology and impact of scarring after burn injury: a systematic review of the literature. J Burn Care Res.

[20] Panayi AC, Heyland DK, Stoppe C (2024). The long-term intercorrelation between post-burn pain, anxiety, and depression: a post hoc analysis of the "RE-ENERGIZE" double-blind, randomized, multicenter placebo-controlled trial. Crit Care.

[21] Dalal PK, Saha R, Agarwal M (2010). Psychiatric aspects of burn. Indian J Plast Surg.

[22] Gerrits MM, van Oppen P, Leone SS, van Marwijk HW, van der Horst HE, Penninx BW (2014). Pain, not chronic disease, is associated with the recurrence of depressive and anxiety disorders. BMC Psychiatry.

[23] Farzan R, Hossein-Nezhadi M, Toloei M, Rimaz S, Ezani F, Jafaryparvar Z (2023). Investigation of anxiety and depression predictors in burn patients hospitalized at Velayat hospital, a newly established burn center. J Burn Care Res.

[24] Edwards RR, Smith MT, Klick B (2007). Symptoms of depression and anxiety as unique predictors of pain-related outcomes following burn injury. Ann Behav Med.

[25] Noorbakhsh SI, Bonar EM, Polinski R, Amin MS (2021). Educational case: Burn injury-pathophysiology, classification, and treatment. Acad Pathol.

[26] Ji RR, Xu ZZ, Gao YJ (2014). Emerging targets in neuroinflammation-driven chronic pain. Nat Rev Drug Discov.

[27] Sapolsky RM (2000). Glucocorticoids and hippocampal atrophy in neuropsychiatric disorders. Arch Gen Psychiatry.

[28] Gatchel RJ, Peng YB, Peters ML, Fuchs PN, Turk DC (2007). The biopsychosocial approach to chronic pain: scientific advances and future directions. Psychol Bull.

[29] Borsook D, Maleki N, Becerra L, McEwen B (2012). Understanding migraine through the lens of maladaptive stress responses: a model disease of allostatic load. Neuron.

[30] Sullivan MJ, Thorn B, Haythornthwaite JA, Keefe F, Martin M, Bradley LA, Lefebvre JC (2001). Theoretical perspectives on the relation between catastrophizing and pain. Clin J Pain.

